# Short and long term exposure to air pollution increases the risk of ischemic heart disease

**DOI:** 10.1038/s41598-021-84587-x

**Published:** 2021-03-03

**Authors:** So Young Kim, Sang Hoon Kim, Jee Hye Wee, Chanyang Min, Sang-Min Han, Seungdo Kim, Hyo Geun Choi

**Affiliations:** 1 Department of Otorhinolaryngology-Head and Neck Surgery, CHA Bundang Medical Center, CHA University, Seongnam, South Korea; 2 Department of Internal Medicine, CHA Bundang Medical Center, CHA University, Seongnam, South Korea; 3 Department of Otorhinolaryngology-Head and Neck Surgery, Hallym University College of Medicine, Anyang, South Korea; 4 Hallym Data Science Laboratory, Hallym University College of Medicine, Anyang, South Korea; 5 Graduate School of Public Health, Seoul National University, Seoul, South Korea; 6 Political Science (Climate and Environmental Policy), Graduate School of Global Cooperation, Hallym University, Chuncheon, South Korea; 7 Research Center for Climate Change and Energy, Hallym University, Chuncheon, South Korea; 8 Hallym Institute for Environmental Diseases (HIED), Chuncheon, South Korea

**Keywords:** Cardiology, Medical research

## Abstract

Previous studies have suggested an increased risk of ischemic heart disease related to air pollution. This study aimed to explore both the short-term and long-term effects of air pollutants on the risk of ischemic heart disease after adjusting for meteorological factors. The Korean National Health Insurance Service-Health Screening Cohort from 2002 to 2013 was used. Overall, 2155 participants with ischemic heart disease and 8620 control participants were analyzed. The meteorological data and air pollution data, including SO_2_ (ppm), NO_2_ (ppm), O_3_ (ppm), CO (ppm), and particulate matter (PM)_10_ (μg/m^3^), were analyzed using conditional logistic regression. Subgroup analyses were performed according to age, sex, income, and region of residence. One-month exposure to SO_2_ was related to 1.36-fold higher odds for ischemic heart disease (95% confidence interval [95% CI] 1.06–1.75). One-year exposure to SO_2_, O_3_, and PM_10_ was associated with 1.58- (95% CI 1.01–2.47), 1.53- (95% CI 1.27–1.84), and 1.14 (95% CI 1.02–1.26)-fold higher odds for ischemic heart disease. In subgroup analyses, the ≥ 60-year-old group, men, individuals with low income, and urban groups demonstrated higher odds associated with 1-month exposure to SO_2_. Short-term exposure to SO_2_ and long-term exposure to SO_2,_ O_3_, and PM_10_ were related to ischemic heart disease.

## Introduction

Ischemic heart disease is a fatal disease with high morbidity and mortality. The prevalence increased from the early twentieth century to the 1960s, likely due to the increase in smoking and high fat intake, which promote the development of coronary atherosclerosis^1^. Although the prevalence and mortality of ischemic heart disease have decreased, it is still one of the leading causes of mortality worldwide^2,3^. Atherosclerotic and calcified plaques in coronary arteries have been described as the main pathologies of ischemic heart disease^4^. Inflammation in the cardiovascular system has been suggested to cause these changes in coronary vessels and to be linked with systemic inflammatory diseases^5^.

Although early diagnosis and intervention increase the survival rate of ischemic heart disease, the primary prevention of ischemic heart disease might be most effective at reducing disease burden. Several modifiable risk factors have been reported, including the lifestyle factors of obesity, alcohol consumption, and tobacco smoking^6–8^. In addition to these lifestyle factors, which are largely dependent on individuals, an accumulating number of studies has documented that environmental factors, including toxic compounds, could be modifiable factors to prevent the risk of ischemic heart disease at the social level^9^.

Many previous epidemiologic studies have described an increased risk of ischemic heart disease related to air pollution^10–16^, particularly long-term exposure to air pollution^11,17^. On the other hand, the effect of short-term exposure to air pollution has also been suggested, with some conflicting findings^10,18^. A case-crossover study reported that a 10 µg/m^3^ increase in exposure to fine particulate pollution (particulate matter with an aerodynamic diameter ≤ 2.5 µm; PM_2.5_) was associated with a 4.5% increased risk of ischemic heart disease (95% CI 1.1–8.0)^10^. However, a time-series study demonstrated that increased exposure to sulfur dioxide (SO_2_), but not PM_2.5_ or nitrogen dioxide (NO_2_), for 3 days was related to an increased risk of ischemic heart disease mortality^18^. The heterogeneous study design, regional and ethnic differences and types of measured pollutants might have contributed to these controversial results.

Few studies have reported a wide range of air pollution exposure periods from short-term to long-term exposure. Moreover, when exploring the effect of air pollution, meteorological factors should be concurrently considered because the concentration and composition of air pollutants might be influenced by these factors, and the risk of cardiovascular disease could be associated with meteorological factors, such as ambient temperature^19^. For instance, the solubility of air pollutants is increased at lower temperatures, and the photolysis reaction could change the composition of air pollutants. Therefore, this study analyzed the effect of air pollutants on ischemic heart disease according to exposure periods prior to the development of ischemic heart disease. To evaluate this effect, we calculated the mean levels of air pollutants for time periods from 3 to 730 days of exposure. To minimize the confounding effects of meteorological factors, they were concurrently analyzed for their association with ischemic heart disease.

## Materials and methods

### Participant selection

This study was approved by the Ethics Committee of Hallym University (2017-I102) and was exempt from requiring informed consent^20^. All procedures were followed in accordance with the relevant guidelines. The Korean National Health Insurance Service-Health Screening Cohort (NHIS-HEALS), meteorological, and air pollution data were used (S1 description)^20–22^.

Participants with ischemic heart disease were selected from 514,866 patients with 497,931,549 medical claim codes (n = 2239)^20–22^. Among these participants, individuals were excluded if they were diagnosed with ischemic heart disease before 2004 (n = 80) to track previous exposure to meteorological factors in the last 2 years. The control group included those without a history of ischemic heart disease from 2002 through 2013 (n = 512,627)^20–22^. The control participants were 1:4 matched for age, sex, income, region of residence, and index date^20^.

**Figure 1:**
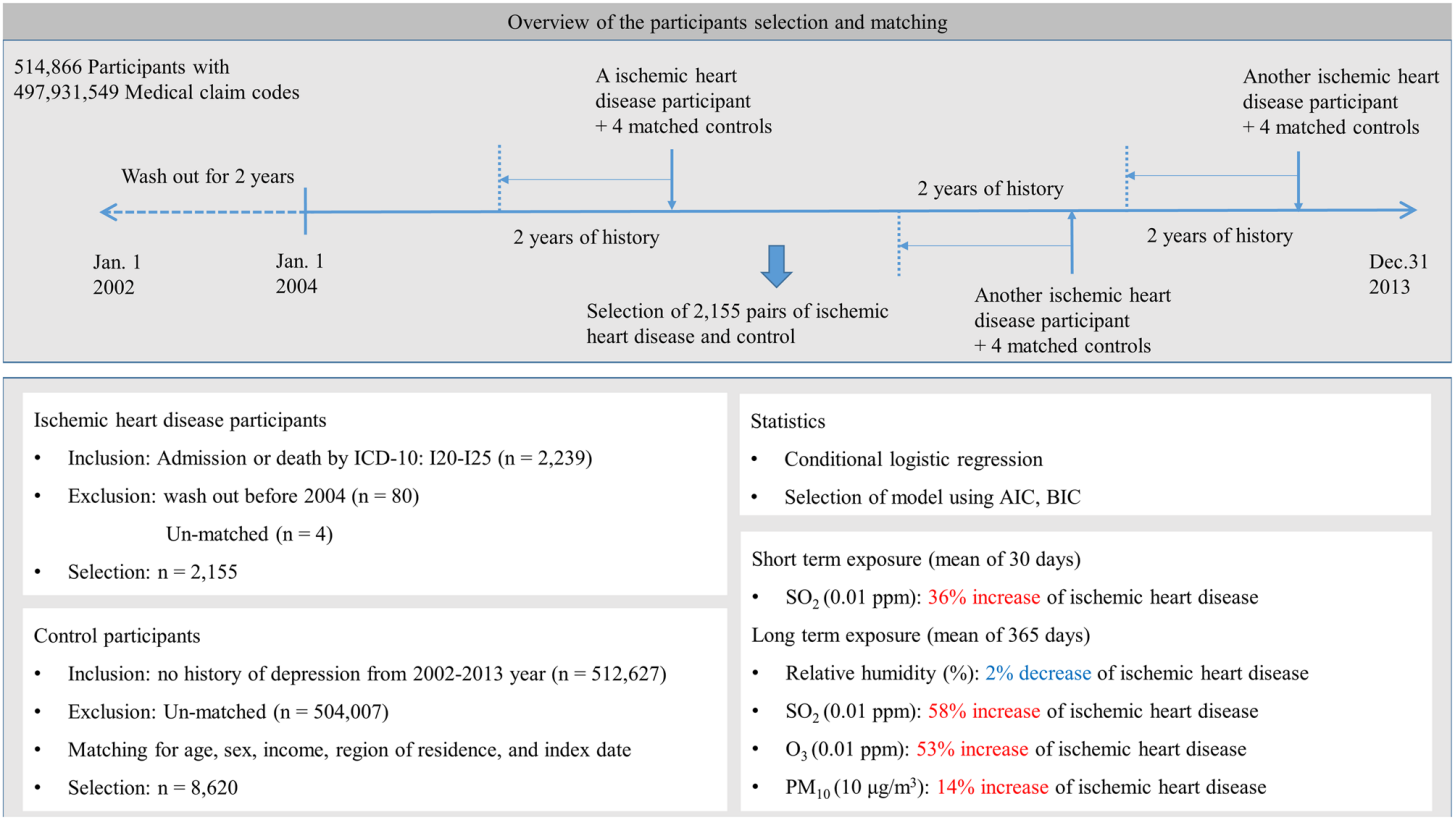
A schematic illustration of the participant selection process used in the present study. Of 1,125,691 participants, 2155 ischemic heart disease participants were matched with 8620 control participants for age, group, sex, income group, and region of residence. Then, ischemic heart disease patients and control participants were matched according to the meteorological data and air pollution data before the index date.

The index date of ischemic heart disease participants was set as the time of diagnosis of ischemic heart disease^20–22^. Some ischemic heart disease participants were excluded because there were not enough matched control participants (n = 4). Collectively, 2155 ischemic heart disease participants were 1:4 matched with 8620 control participants (Fig. 1)^20–22^.

We analyzed meteorological and air pollution data over a mean of 3 days, 5 days, 10 days, 15 days, 1 month (30 days), 2 months (60 days), 3 months (90 days), 6 months (180 days), 9 months (270 days), 1 year (365 days), 18 months (540 days), and 2 years (730 days) before the date of diagnosis or the index date in each participant according to their region of residence.

### Variables

#### Independent variables

Daily mean temperature (°C), daily highest temperature (°C), daily lowest temperature (°C), relative humidity (%), ambient atmospheric pressure (hPa), SO_2_ (ppm), NO_2_ (ppm), O_3_ (ppm), CO (ppm), and PM_10_ (μg/m^3^) for 3 days, 5 days, 10 days, 15 days, 1 month (30 days), 2 months (60 days), 3 months (90 days), 6 months (180 days), 9 months (270 days), 1 year (365 days), 18 months (540 days), and 2 years (730 days) were analyzed as independent variables (S1 description)^20^. These data were gathered from Air Korea, which is managed by the Ministry of Environment of Korea^23^. The air pollution and meteorological factors measured at the closest points to the region of residence of participants were applied for analyses.

#### Dependent variable

Ischemic heart disease was classified using ICD-10 codes (I20-I25). Among the participants, those who were hospitalized because of ischemic heart disease or who died because of ischemic heart disease were included^24^.

#### Covariate

Age groups were classified in 5-year intervals: 40–44, 45–49, 50–54…, and 85 + years old. Income groups were divided into 5 classes (class 1 [lowest income]-5 [highest income]). The region of residence was grouped into urban and rural areas^25^.

Tobacco smoking, alcohol consumption, and obesity using BMI (body mass index, kg/m^2^) were included^26^.

The Charlson Comorbidity Index (CCI) was included^27,28^.

### Statistical analyses

The general characteristics of the ischemic heart disease and control groups were compared using the chi-square test^20^. The mean meteorological and air pollution data 30 days and 365 days before the index date were compared using independent t-tests.

To analyze the odds ratios (ORs) with the 95% confidence intervals (CIs) of meteorological and air pollution data for ischemic heart disease participants compared to control participants, a crude model (simple model), adjusted model (all insertion model), and final model (backward selection of variables) were calculated using conditional logistic regression^20^. As ORs of independent variables were calculated as continuous variables, they were displayed as SO_2_ per 0.01 ppm, NO_2_ per 0.1 ppm, O_3_ per 0.01 ppm, CO per 1 ppm, and PM_10_ per 10 μg/m^3^. In these analyses, age, sex, income, and region of residence were stratified. In the analyses of 3 days, 5 days, 10 days, 15 days, 30 days, 60 days, 90 days, 180 days, 270 days, 365 days, 540 days, and 730 days of exposure, we selected 30 days as the short-term exposure and 365 days as the long-term exposure^20^. The results of other days of exposure are displayed in the supplemental file (Supplementary Tables S2–S12). To select final models, Akaike information criterion and Baysian information criterion of air pollutants were analyzed (Supplementary Table S13). The correlations between meteorological and air pollutants were provided as supplemental tables (Supplementary Tables S14–S15).

For the subgroup analysis, we divided participants by age, sex, income, and region (< 60 years old and ≥ 60 years old; men and women; low income [income 1–3] and high income [income 4–5]; urban and rural, respectively) in the final model^20^.

Two-tailed analyses were performed, and significance was defined as P values less than 0.05. SAS version 9.4 (SAS Institute Inc., Cary, NC, USA) was used for statistical analyses^20^.

## Results

Regarding meteorological and air pollution data, SO_2_ for 30 days, temperature (mean, highest, and lowest), relative humidity, SO_2_, NO_2_ and CO for 365 days were different between the ischemic heart disease group and the control group (all P < 0.05, Table 1).

**Table 1:** General characteristics of the participants.

Characteristics	Total participants		
	IHD	Control	P-value
**Age (years old, n, %)**			1.000
40–44	12 (0.6)	48 (0.6)	
45–49	62 (2.9)	248 (2.9)	
50–54	139 (6.5)	556 (6.5)	
55–59	151 (7.0)	604 (7.0)	
60–64	176 (8.2)	704 (8.2)	
65–69	315 (14.6)	1260 (14.6)	
70–74	408 (18.9)	1632 (18.9)	
75–79	397 (18.4)	1588 (18.4)	
80–84	364 (16.9)	1456 (16.9)	
85 +	131 (6.1)	524 (6.1)	
**Sex (n, %)**			1.000
Male	1447 (67.2)	5788 (67.2)	
Female	708 (32.9)	2832 (32.9)	
**Income (n, %)**			1.000
1 (lowest)	475 (22.0)	1900 (22.0)	
2	304 (14.1)	1216 (14.1)	
3	308 (14.3)	1232 (14.3)	
4	411 (19.1)	1644 (19.1)	
5 (highest)	657 (30.5)	2628 (30.5)	
**Region of residence (n, %)**			1.000
Urban	831 (38.6)	3324 (38.6)	
Rural	1324 (61.4)	5296 (61.4)	
**Charlson comorbidity index (n, %)**			< 0.001*
0	1938 (89.9)	8352 (96.9)	
1	34 (1.6)	26 (0.3)	
2	41 (1.9)	41 (0.5)	
3	34 (1.6)	33 (0.4)	
≥ 4	108 (5.0)	168 (2.0)	
**Obesity (BMI, kg/m**^**2**^**, n, %)**			< 0.001*
< 18.5 (underweight)	143 (6.6)	376 (4.4)	
≥ 18.5 to < 23 (normal)	833 (38.7)	3290 (38.2)	
≥ 23 to < 25 (overweight)	511 (23.7)	2224 (25.8)	
≥ 25 to < 30 (obese I)	604 (28.0)	2512 (29.1)	
≥ 30 (obese II)	64 (3.0)	218 (2.5)	
**Smoking status (n, %)**			< 0.001*
Nonsmoker	1310 (60.8)	5746 (66.7)	
Past smoker	262 (12.2)	1242 (14.4)	
Current smoker	583 (27.1)	1632 (18.9)	
**Alcohol consumption (n, %)**			< 0.001*
< 1 time a week	1682 (78.1)	6412 (74.4)	
≥ 1 time a week	473 (22.0)	2208 (25.6)	
**Meteorological and air pollution data (mean, SD)**			
Mean temperature for 30 days (°C)	12.2 (9.5)	12.1 (9.5)	0.868
Highest temperature for 30 days (°C)	17.4 (9.3)	17.4 (9.3)	0.937
Lowest temperature for 30 days (°C)	7.7 (9.9)	7.6 (9.9)	0.805
Relative humidity for 30 days (%)	65.2 (10.1)	65.4 (10.0)	0.308
Ambient atmospheric pressure for 30 days (hPa)	1006.3 (7.5)	1006.3 (7.4)	0.707
SO_2_ for 30 days (ppb)	5.7 (1.9)	5.6 (1.9)	0.016*
NO_2_ for 30 days (ppb)	23.5 (8.1)	23.8 (8.2)	0.129
O_3_ for 30 days (ppb)	23.3 (8.2)	23.1 (8.3)	0.243
CO for 30 days (ppb)	571.4 (182.4)	571.0 (179.3)	0.940
PM_10_ for 30 days (μg/m^3^)	52.0 (14.5)	51.9 (14.8)	0.862
Mean temperature for 365 days (°C)	12.9 (1.3)	12.8 (1.2)	0.004*
Highest temperature for 365 days (°C)	18.1 (1.1)	18.0 (1.1)	0.009*
Lowest temperature for 365 days (°C)	8.4 (1.8)	8.3 (1.7)	0.009*
Relative humidity for 365 days (%)	65.7 (4.5)	65.9 (4.5)	0.020*
Ambient atmospheric pressure for 365 days (hPa)	1005.7 (4.6)	1005.8 (4.5)	0.435
SO_2_ for 365 days (ppb)	5.6 (1.1)	5.5 (1.2)	0.004*
NO_2_ for 365 days (ppb)	23.3 (6.5)	23.7 (6.7)	0.021*
O_3_ for 365 days (ppb)	23.7 (3.7)	23.3 (3.7)	< 0.001*
CO for 365 days (ppb)	567.0 (110.2)	570.4 (105.2)	0.201
PM_10_ for 365 days (μg/m^3^)	52.3 (7.2)	52.2 (7.2)	0.713

*IHD* ischemic heart disease, *BMI* body mass index (kg/m^2^), *ppb* parts per billion, *ppm* part per million (= 1000 ppb), *SD* standard deviation.

*Chi-square test or independent t-test. Significance at P < 0.05.

**Figure 2:**
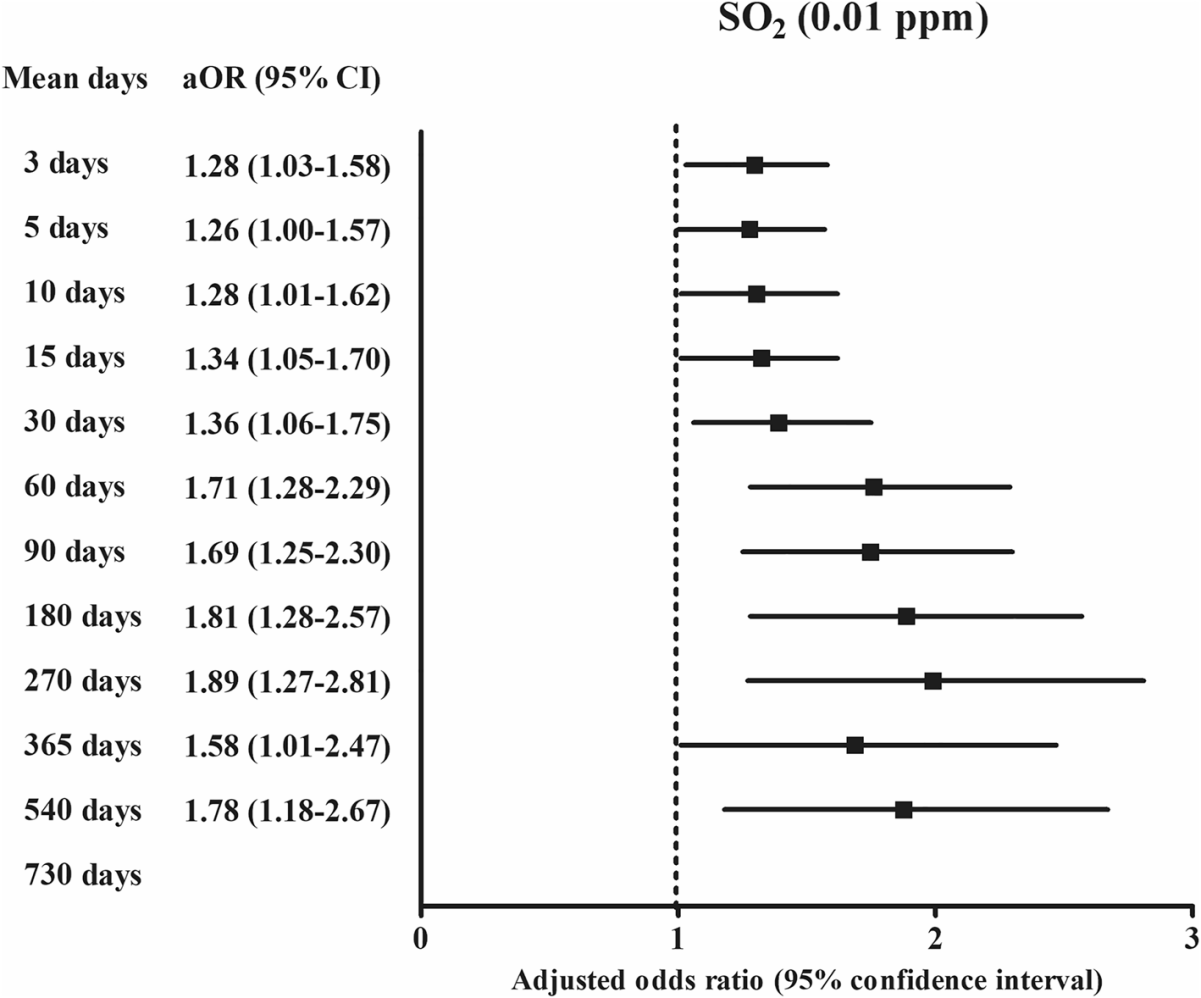
The odds ratios (95% confidence interval) of SO_2_ (0.01 ppm) for 3 days, 5 days, 10 days, 15 days, 30 days, 60 days, 90 days, 180 days, 270 days, 365 days, and 540 days for ischemic heart disease.

For 30 days of exposure, the OR was 1.36 (95% CI 1.06–1.75, Fig. 2) for SO_2_ (0.01 ppm) in the ischemic heart disease group compared with the control group using the final model (Table 2).

**Table 2:** Crude and adjusted odd ratios (95% confidence interval) of the meteorological and pollution matter (mean of 30 days before the index date) for ischemic heart disease.

Characteristics	Odds ratio for ischemic heart disease (95% CI)					
	Crude^a^	P-value	Adjusted^a,b^	P-value	Final^a,c^	P-value
Mean temperature for 30 days (°C)	1.00 (1.00–1.01)	0.864	0.90 (0.65–1.24)	0.506		
Highest temperature for 30 days (°C)	1.00 (1.00–1.01)	0.935	1.04 (0.89–1.21)	0.660		
Lowest temperature for 30 days (°C)	1.00 (1.00–1.01)	0.798	1.09 (0.92–1.29)	0.334		
Relative humidity for 30 days (%)	1.00 (0.99–1.00)	0.271	0.99 (0.98–1.00)	0.045*		
Ambient atmospheric pressure for 30 days (hPa)	1.00 (0.99–1.01)	0.697	1.00 (0.99–1.01)	0.923		
SO_2_ for 30 days (0.01 ppm)	1.36 (1.07–1.75)	0.014*	1.74 (1.18–2.56)	0.005*	1.36 (1.06–1.75)	0.015*
NO_2_ for 30 days (0.1 ppm)	0.59 (0.31–1.11)	0.099	0.40 (0.15–1.07)	0.066		
O_3_ for 30 days (0.01 ppm)	1.04 (0.98–1.10)	0.227	0.98 (0.89–1.09)	0.727		
CO for 30 days (ppm)	1.01 (0.77–1.32)	0.939	1.02 (0.61–1.73)	0.935		
PM_10_ for 30 days (10 μg/m^3^)	1.00 (0.97–1.04)	0.859	1.01 (0.96–1.07)	0.593		

*Conditional logistic regression model, Significance at P < 0.05.

^a^Stratified model for age, sex, income, and region of residence.

^b^Adjusted model was adjusted for obesity, smoking status (current smoker compared to nonsmoker or past smoker), frequency of alcohol consumption (≥ 1 time a week compared to < 1 time a week), CCI score, mean temperature, highest temperature, lowest temperature, relative humidity, atmospheric pressure, SO_2_, NO_2_, O_3_, CO, and PM_10_.

^c^Final model was adjusted for obesity, smoking status (current smoker compared to nonsmoker or past smoker), frequency of alcohol consumption (≥ 1 time a week compared to < 1 time a week), CCI score, relative humidity, SO_2_, NO_2_, O_3_, CO, and PM_10_ using the backward selection method.

**Figure 3:**
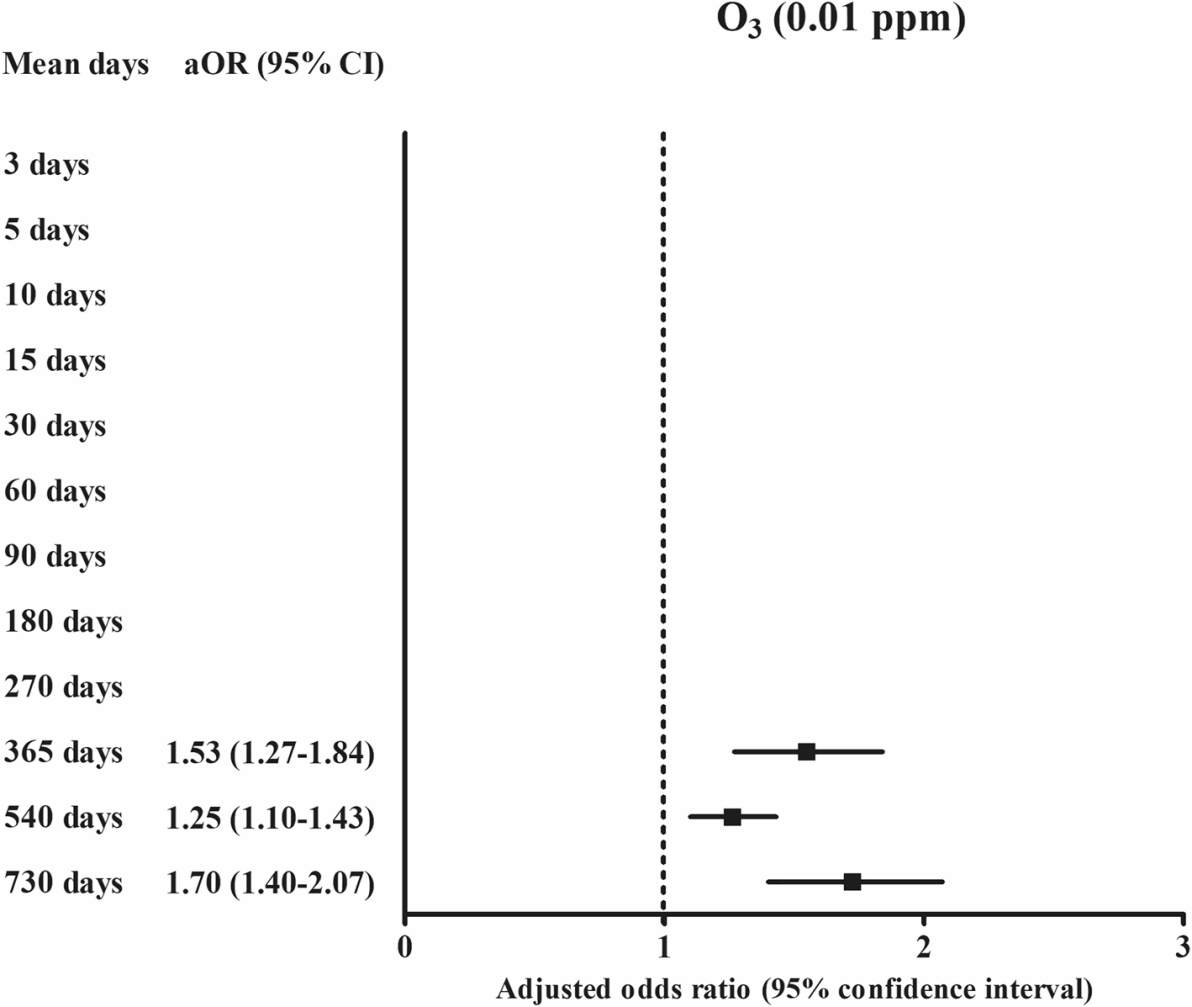
The odds ratios (95% confidence interval) of O_3_ (0.01 ppm) for 365 days, 540 days, and 730 days for ischemic heart disease.

**Figure 4:**
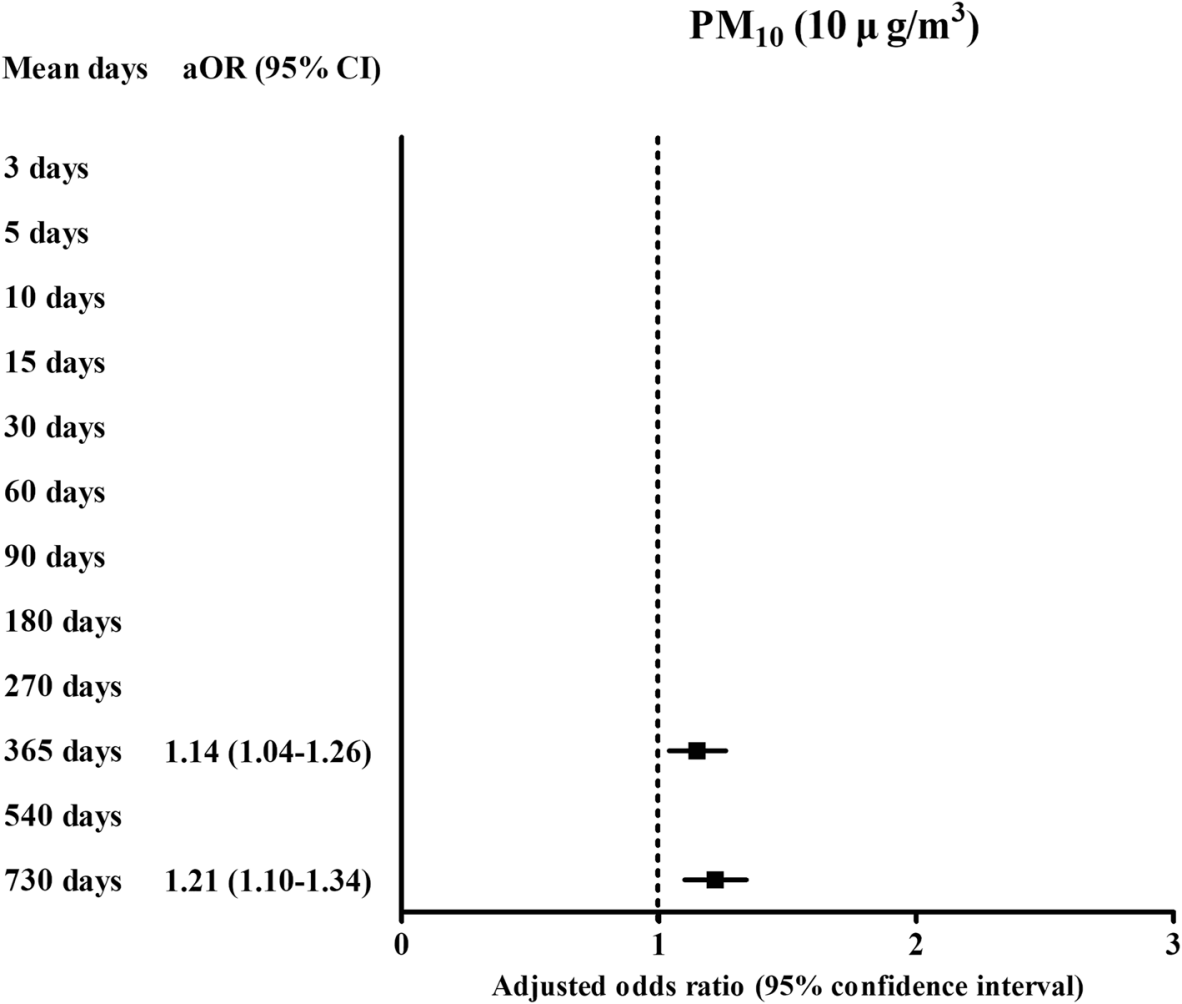
The odds ratios (95% confidence interval) of PM_10_ (10 μg/m^3^) for 365 days and 730 days for ischemic heart disease.

For one-year exposure, the ORs were 0.98 (95% CI 0.96–0.99) for relative humidity (%), 1.58 (95% CI 1.01–2.47, Fig. 2) for SO_2_ (0.01 ppm), 1.53 (95% CI 1.27–1.84, Fig. 3) for O_3_ (0.01 ppm), and 1.14 (95% CI 1.02–1.26, Fig. 4) for PM_10_ (10 μg/m^3^) in the ischemic heart disease group compared with the control group using the final model (Table 3).

**Table 3:** Crude and adjusted odd ratios (95% confidence interval) of the meteorological and pollution matter (mean of 365 days before the index date) for ischemic heart disease.

Characteristics	Odds ratio for ischemic heart disease (95% CI)					
	Crude^a^	P-value	Adjusted^a,b^	P-value	Final^a,c^	P-value
Mean temperature for 365 days (°C)	1.07 (1.03–1.12)	0.001*	0.89 (0.40–1.96)	0.768		
Highest temperature for 365 days (°C)	1.06 (1.02–1.11)	0.009*	1.00 (0.70–1.43)	0.989		
Lowest temperature for 365 days (°C)	1.06 (1.02–1.10)	0.001*	1.09 (0.72–1.66)	0.689		
Relative humidity for 365 days (%)	0.98 (0.96–0.99)	0.002*	0.97 (0.95–0.99)	0.001*	0.98 (0.96–0.99)	0.002*
Ambient atmospheric pressure for 365 days (hPa)	1.00 (0.99–1.01)	0.413	1.01 (0.99–1.02)	0.516		
SO_2_ for 365 days (0.01 ppm)	1.84 (1.22–2.77)	0.004*	1.88 (1.12–3.18)	0.017*	1.58 (1.01–2.47)	0.045*
NO_2_ for 365 days (0.1 ppm)	0.35 (0.16–0.79)	0.011*	0.18 (0.04–0.90)	0.037*		
O_3_ for 365 days (0.01 ppm)	1.31 (1.15–1.50)	< 0.001*	1.27 (0.98–1.64)	0.070	1.53 (1.27–1.84)	< 0.001*
CO for 365 days (ppm)	0.73 (0.46–1.15)	0.174	0.81 (0.38–1.70)	0.569		
PM_10_ for 365 days (10 μg/m^3^)	1.01 (0.95–1.09)	0.701	1.19 (1.12–3.18)	0.001*	1.14 (1.04–1.26)	0.007*

*Conditional logistic regression model, Significance at P < 0.05.

^a^Stratified model for age, sex, income, and region of residence.

^b^Adjusted model was adjusted for obesity, smoking status (current smoker compared to nonsmoker or past smoker), frequency of alcohol consumption (≥ 1 time a week compared to < 1 time a week), CCI score, mean temperature, highest temperature, lowest temperature, relative humidity, atmospheric pressure, SO_2_, NO_2_, O_3_, CO, and PM_10_.

^c^Final model was adjusted for obesity, smoking status (current smoker compared to nonsmoker or past smoker), frequency of alcohol consumption (≥ 1 time a week compared to < 1 time a week), CCI score, relative humidity, SO_2_, NO_2_, O_3_, CO, and PM_10_ using the backward selection method.

**Figure 5:**
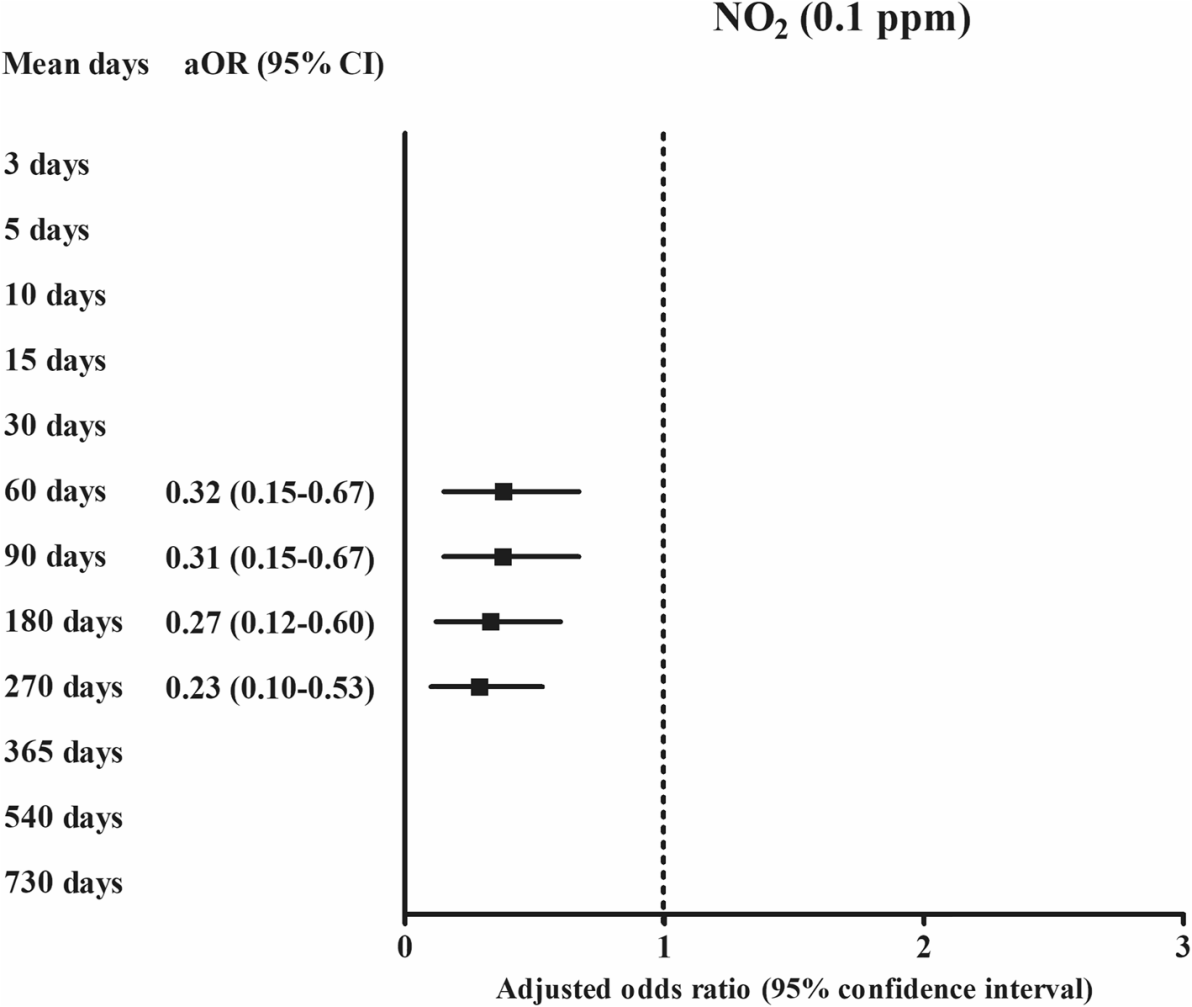
The odds ratios (95% confidence interval) of NO_2_ (0.1 ppm) for 60 days, 90 days, 180 days, and 270 days for ischemic heart disease.

According to the duration of exposures, the exposure of SO_2_ (0.01 ppm) was positively related with ischemic heart disease in 3 days, 5 days, 10 days, 15 days, 30 days, 60 days, 90 days, 180 days, 270 days, 365 days, and 540 days of exposure (Fig. 2 and Supplementary Table S1). For PM_10_ (10 μg/m^3^), 365 days and 730 days of PM_10_ (10 μg/m^3^) exposure were positively related with ischemic heart disease (Fig. 4). For O_3_ (0.01 ppm), 365 days, 540 days, and 730 days of O_3_ (0.01 ppm) exposure were positively related with ischemic heart disease (Fig. 3). The exposure of NO_2_ (0.1 ppm) for 60 days, 90 days, 180 days, and 270 days was negatively related with ischemic heart disease (Fig. 5).

In the subgroup analyses using the final model, in the age groups < 60 years old and ≥ 60 years old, the odds for O_3_ and PM_10_ were higher in ischemic heart disease participants than in the control group after 1 year of exposure. In the age ≥ 60 years group, the odds for SO_2_ were higher in ischemic heart disease participants than in the control group after 30 days and one year of exposure. In men, individuals with low income, and urban groups, the odds for SO_2_ for 30 days of exposure and odds for O_3_ and PM_10_ for one year of exposure were higher in ischemic heart disease patients than in the control group. In the high-income and rural groups, the odds for SO_2_ for one year of exposure were higher in ischemic heart disease patients than in the control group (Table 4).

**Table 4:** Adjusted odd ratios (95% confidence interval) of the meteorological and pollution matter for ischemic heart disease according to age and sex in the final model.

Characteristics	Means of 30 days		Means of 365 days	
	Odd ratios (95% CI)	P-value	Odd ratios (95% CI)	P-value
**Age < 60 years old (n = 1820)**				
O_3_ (0.01 ppm)			2.21 (1.46–3.34)	< 0.001*
PM_10_ (10 μg/m^3^)			1.28 (1.03–1.60)	0.027*
**Age ≥ 60 years old (n = 8955)**				
SO_2_ (0.01 ppm)	1.37 (1.04–1.79)	0.025*	1.67 (1.02–2.72)	0.042*
Relative humidity (%)			0.97 (0.96–0.99)	0.001*
O_3_ (0.01 ppm)			1.42 (1.16–1.75)	0.001*
PM_10_ (10 μg/m^3^)			1.12 (1.01–1.25)	0.032*
**Men (n = 7235)**				
SO_2_ (0.01 ppm)	1.45 (1.07–1.96)	0.016*		
O_3_ (0.01 ppm)			1.54 (1.24–1.92)	< 0.001*
PM_10_ (10 μg/m^3^)			1.19 (1.06–1.32)	0.003*
**Women (n = 3540)**				
Relative humidity (%)			0.95 (0.93–0.98)	< 0.001*
NO_2_ (0.1 ppm)			0.09 (0.02–0.39)	0.002*
**Low income (n = 5435)**				
SO_2_ (0.01 ppm)	1.43 (1.00–2.04)	0.050*		
O_3_ (0.01 ppm)			1.69 (1.32–2.18)	< 0.001*
PM_10_ (10 μg/m^3^)			1.25 (1.10–1.41)	0.001*
**High income (n = 5340)**				
Relative humidity (%)			0.97 (0.95–0.99)	0.001*
SO_2_ (0.01 ppm)			2.09 (1.13–3.88)	0.019*
NO_2_ (0.1 ppm)			0.22 (0.07–0.69)	0.010*
**Urban (n = 4155)**				
SO_2_ (0.01 ppm)	1.62 (1.05–2.49)	0.028*		
Relative humidity (%)			0.98 (0.96–0.99)	0.009*
O_3_ (0.01 ppm)			2.08 (1.60–2.70)	< 0.001*
PM_10_ (10 μg/m^3^)			1.39 (1.21–1.60)	< 0.001*
**Rural (n = 6620)**				
Relative humidity (%)			0.97 (0.95–0.99)	0.008*
SO_2_ (0.01 ppm)			1.71 (1.00–2.93)	0.050*

*Conditional logistic regression model; Significance at P < 0.05.

^a^Stratified model for age, sex, income, and region of residence.

^b^Final model was adjusted for obesity, smoking status (current smoker compared to nonsmoker or past smoker), frequency of alcohol consumption (≥ 1 time a week compared to < 1 time a week), CCI score, relative humidity, SO_2_, NO_2_, O_3_, CO, and PM_10_ using the backward selection method.

## Discussion

Both short-term and long-term exposure to air pollutants were related to ischemic heart disease in the present study. The types of air pollutants that impacted ischemic heart disease differed according to exposure period. Short-term (30 days) exposure to SO_2_ was related to higher odds of ischemic heart disease. For long-term (365 days) exposure, higher levels of SO_2_, O_3_, and PM_10_ were associated with ischemic heart disease. In addition, the effects of air pollutants on ischemic heart disease were different according to the demographic factors of age and sex and the socioeconomic factors of income level and region of residence.

Short-term exposure to SO_2_ was associated with higher odds of ischemic heart disease in this study. In line with the present results, previous studies have reported elevated mortality related to short-term exposure to SO_2_^18,29^. Although some prior studies demonstrated an association between exposure to PM_10_, SO_2_, and NO_2_ with the disease burden of ischemic heart disease (years of life lost), one time-series study reported that SO_2_, but not other air pollutants or PMs, was related to the increased mortality of ischemic heart disease (excess risk of death = 3.18%, 95% CI 1.19–5.17)^18^. They found that gaseous pollutants, such as SO_2_, had higher impacts on the risk of ischemic heart disease than PMs^18^. Oxidative stress and the inflammatory response have been suggested as possible pathophysiologic mechanisms for the impact of SO_2_ on ischemic heart disease^30^. Sulfate exposure for 2–7 days was associated with oxidative stress markers of urinary creatinine-indexed 8-epi-prostaglandin F2α in the Framingham heart study^30^.

Short-term exposure to other air pollutants, including NO_2_ and PM_10,_ did not show an association with ischemic heart disease in the present study. Previous epidemiologic studies have suggested that the source or components of PMs are crucial for their hazardous impact on ischemic heart disease^11^. The PM_2.5_ from wind-blown soil or biomass combustion was not associated with ischemic heart mortality^11^. The present study could not differentiate the sources of PM_10_ because its heterogeneous composition could attenuate its adverse impacts on ischemic heart disease.

Long-term exposure to SO_2_, O_3_, and PM_10_ was related to higher odds of ischemic heart disease in this study. A large amount of previous epidemiologic data supports the long-term effects of air pollutants on the risk of ischemic heart disease^31,32^. The mortality of ischemic heart disease was 1.03-fold higher in patients who were exposed to a high level of PM_2.5_ in the form of diesel traffic-related elemental carbon (95% CI 1.00–1.06) from 2000 to 2005 in the US^11^. Multiple pathophysiologic mechanisms, including the systemic inflammatory response, prothrombotic pathway activation, oxidative stress, vascular dysfunction and remodeling, autonomic dysfunction, and epigenetic factors, have been proposed to mediate the impact of air pollutants on ischemic heart disease^12^. For instance, coronary artery calcification was proposed as one of the pathophysiologic mechanisms for the effect of air pollutants, including PMs and O_3,_ on the risk of ischemic heart disease^24,33^. The coronary artery calcium score, which is considered an atherosclerotic marker, was associated with elevated levels of PM_2.5_ (27.2%, 95% CI 10.8–46.1%)^31^. Although the pathophysiologic mechanism of the effect of O_3_ on ischemic heart disease remains elusive, oxidizing activities could induce inflammation in the coronary artery, which might result in atherosclerotic plaque formation and narrowing of the arterial lumen with increasing wall thickness^32^. To support this hypothesis, it was reported that long-term exposure to O_3_ was related to increased thickness of the common carotid artery (5.6 µm, 95% CI 1.4–9.7) and carotid plaque burden (OR 1.2, 95% CI 1.1–1.4)^32^.

The relative humidity for one year was negatively associated with ischemic heart disease in this study. Previous studies have suggested the contributions of meteorological factors of temperature variability with the risk of ischemic heart disease, although no prior research investigated the association of humidity with ischemic heart disease^34,35^. The potential impact of humidity on the solubility of gaseous pollutants and moisture content, which may decrease the amount of air pollutant exposure, could mediate the decreased rate of ischemic heart disease in high humidity conditions.

The impacts of air pollutants were prominent in the old age group and men in this study. Previous studies have also suggested the higher susceptibility of older populations and men to the impacts of air pollutants on ischemic heart disease^18,31^. Preformed or subclinical atherosclerotic changes of the coronary artery could more easily progress due to the impacts of air pollutants on inflammation and atherosclerosis, although the synergistic or additive effects of air pollutants could not be determined in the current study. The high prevalence of ischemic heart disease in men compared to that in women could strengthen the statistical power in this population. In addition, populations with poor socioeconomic status showed a relationship between short-term exposure to SO_2_ and ischemic heart disease in this study. Several previous studies investigated socioeconomic disparities and the impact of a higher burden of air pollution on morbidities and mortalities in minorities, although the results had some heterogeneity according to the air pollution models^36,37^. Populations with poor socioeconomic status have been reported to be exposed to more air pollution because of the lack of availability of air conditioning and increased industrial exposure^38–41^. In addition to high exposure to air pollution, poor socioeconomic groups were reported to have increased susceptibility to air pollution due to underlying health statuses and reduced access to medical care^38^. The association of short-term exposure to SO_2_ and ischemic heart disease in the urban subgroup might be attributed to the higher level of air pollution in urban areas than in rural areas.

The analysis of a large, representative national cohort population strengthened the statistical power of the present study. The large study population enabled the selection of a control population that was matched for age, sex, income, and region of residence. Possible confounders were comprehensively considered in this study. In addition to past medical histories, lifestyle factors of smoking, alcohol consumption, and obesity were adjusted. Moreover, meteorological factors were concurrently considered along with air pollutants. The meteorological factors and air pollution data were collected and validated by the Korea Meteorological Administration. Based on these verified data, this study investigated both the short-term and long-term effects of air pollution on ischemic heart disease. However, a few limitations existed in the current study. Possible collinearity between air pollutants might exist, although we adjusted for these variables in the final models (Supplementary Tables S14–S15). The level of exposure to PM_2.5_ was not available in this cohort. Because the exposure to air pollutants was based on the registered region of residence, the migration of participants during follow-up periods could not be accounted for in the present study. In addition, indoor exposure to air pollutants could not be individually assessed. For the diagnosis of ischemic heart disease, we could not differentiate the types or severity of disease because this study was based on health claims data. Last, because this study was based on Koreans, ethnic differences should be considered when interpreting this study.

## Conclusions

Short-term exposure to SO_2_ and long-term exposure to SO_2_, O_3_, and PM_10_ were associated with an increased risk of ischemic heart disease. The older, male, low-income, and urban groups demonstrated an apparent association between short-term exposure to SO_2_ and ischemic heart disease.

## Supplementary Information

Supplementary Information.

## Data Availability

Releasing of the data by the researcher is not allowed legally. All data are available from the database of the National health Insurance Sharing Service (NHISS; https://nhiss.nhis.or.kr/). NHISS allows data access, at a particular cost, for any researcher who promises to follow the research ethics. Data of this article can be downloaded from the website after promising to follow the research ethics.
