# Supplementary material for: Short and long term exposure to air pollution increases the risk of ischemic heart disease
Source: Sci Rep. 2021 Mar 3;11:5108. doi: 10.1038/s41598-021-84587-x (PMC7930275; doi:10.1038/s41598-021-84587-x)
Supplement: Supplementary file 1 — Supplementary Information. [file 41598_2021_84587_MOESM1_ESM.docx]

**Short and long term exposure to air pollution increases the risk of ischemic heart disease**

So Young Kim, MD, PhD^1^, Sang Hoon Kim, MD, PhD ^2^, Jee Hye Wee, MD, PhD ^3^, Chanyang Min, PhD^4,5^, Sang-Min Han, PhD^6^, Seungdo Kim, PhD^7^, Hyo Geun Choi, MD, PhD ^3,4,8*^

^1^Department of Otorhinolaryngology-Head & Neck Surgery, CHA Bundang Medical Center, CHA University, Seongnam, Korea

^2^Department of Internal medicine, CHA Bundang Medical Center, CHA University, Seongnam, Korea

^3^Department of Otorhinolaryngology-Head & Neck Surgery, Hallym University College of Medicine, Anyang, Korea

^4^Hallym Data Science Laboratory, Hallym University College of Medicine, Anyang, Korea

^5^Graduate School of Public Health, Seoul National University, Seoul, Korea

^6^Political Science (Climate and Environmental Policy), Graduate School of Global Cooperation, Hallym University, Chuncheon, Korea

^7^Research Center for Climate Change and Energy, Hallym University, Chuncheon, Korea

^8^Hallym Institute for Environmental Diseases (HIED), Chuncheon, Korea

**Word counts:** 2,950

**Running title**: Air pollution and ischemic heart disease

***Correspondence:** Hyo Geun Choi [pupen@naver.com](mailto:pupen@naver.com)

## **S1 Descriptions: Study Population and Data Collection**

This national cohort study relied on data from the Korean National Health Insurance Service-Health Screening Cohort (NHIS-HEALS) [[11](#_ENREF_11)]. The Korean National Health Insurance Service (NHIS) randomly selects approximately 10% of individuals who underwent health examinations from 2002 to 2003 (n = ~515,000) directly from the entire population (n = ~5,150,000). Age and sex specific distributions of the cohort population are described online [[12](#_ENREF_12)]. The details of the methods used to perform these procedures are provided by the National Health Insurance Sharing Service [[13](#_ENREF_13)].

All insured Koreans who are at least 40 years old and their dependents undergo no-cost biannual health examinations [[14](#_ENREF_14)]. Each examinee must complete a standard questionnaire in for this health screening program [[14](#_ENREF_14)]. Because all Korean citizens are recognized by a 13-digit resident registration number from birth to death, exact population statistics can be determined using this database. It is mandatory for all Koreans to enroll in the NHIS. All Korean hospitals and clinics use the 13-digit resident registration number to register individual patients in the medical insurance system. Therefore, the risk of overlapping medical records is minimal, even if a patient moves from one place to another. Moreover, all medical treatments in Korea can be tracked without exception using the Korean Health Insurance Review & Assessment (HIRA) system. In Korea, providing a notice of death to an administrative entity is legally required before a funeral can be held, and the cause and date of death are recorded by medical doctors on a death certificate.

This cohort database includes (i) personal information, (ii) health insurance claim codes (procedures and prescriptions), (iii) diagnostic codes using the International Classification of Disease-10 (ICD-10), (iv) death records from the Korean National Statistical Office (using the Korean Standard Classification of disease), (v) socioeconomic data (residence and income), (vi) medical examination data (vii) health examination data (body mass index [BMI], drinking and smoking habits, blood pressure, urinalysis, hemoglobin, fasting glucose, lipid parameters, creatinine, and liver enzymes) for each participant over the period from 2002 to 2013 [[13](#_ENREF_13), [14](#_ENREF_14)].

**Meteorological Data**

Temperature (˚C), Relative humidity (%), were Spot atmospheric pressure (hPa) data were obtained from the meteorological administration. It was measured by automated synoptic observing system (ASOS) and manually in 94 places hourly. Quality was controlled following quality inspection manual (<https://data.kma.go.kr/cmmn/main.do>).

SO_2_ (ppm), NO_2_ (ppm), O_3_ (ppm), CO (ppm), and PM_10_ (μg/m^3^) data were obtained by the ministry of environment. It was measured by ASOS in 273 place over the country hourly. Quality was controlled following air pollution quality control manual (<http://www.me.go.kr/home/web/index.do?menuId=10259>). We used daily mean values.

**Table S2** Crude and adjusted odd ratios (95% confidence interval) of the meteorological and pollution matter (mean of 3 days before index date) for ischemic heart disease

| Characteristics | | Odds ratio for ischemic heart disease (95% CI) | | | | | |
| --- | --- | --- | --- | --- | --- | --- | --- |
|  |  | Crude † | P-value | Adjusted †‡ | P-value | Final †§ | P-value |
| Mean temperature for 3 days (˚C) | | 1.00 (1.00-1.01) | 0.848 | 0.98 (0.84-1.13) | 0.765 |  |  |
| Highest temperature for 3 days (˚C) | | 1.00 (1.00-1.01) | 0.835 | 1.01 (0.94-1.09) | 0.726 |  |  |
| Lowest temperature for 3 days (˚C) | | 1.00 (1.00-1.01) | 0.849 | 1.01 (0.94-1.10) | 0.756 |  |  |
| Relative humidity for 3 days (%) | | 1.00 (0.99-1.00) | 0.211 | 1.00 (0.99-1.00) | 0.242 |  |  |
| Ambient atmospheric pressure for 3 days (hPa) | | 1.00 (0.99-1.00) | 0.621 | 1.00 (0.99-1.01) | 0.417 |  |  |
| SO_2_ for 3 days (0.01 ppm) | | 1.28 (1.03-1.58) | 0.023* | 1.72 (1.27-2.34) | 0.001* | 1.28 (1.03-1.58) | 0.025* |
| NO_2_ for 3 days (0.1 ppm) | | 0.68 (0.41-1.14) | 0.146 | 0.54 (0.25-1.21) | 0.136 |  |  |
| O_3_ for 3 days (0.01 ppm) | | 1.02 (0.98-1.08) | 0.339 | 0.96 (0.90-1.04) | 0.313 |  |  |
| CO for 3 days (ppm) | | 0.95 (0.76-1.19) | 0.663 | 0.82 (0.54-1.24) | 0.352 |  |  |
| PM_10_ for 3 days (10 μg/m^3^) | | 1.00 (0.98-1.02) | 0.786 | 1.00 (0.97-1.02) | 0.730 |  |  |

* Conditional logistic regression model, Significance at P < 0.05

† Stratified model for age, sex, income, and region of residence.

‡ Adjusted model was adjusted for obesity, smoking status (current smoker compared to nonsmoker or past smoker), frequency of alcohol consumption (≥ 1 time a week compared to < 1 time a week), CCI score, mean temperature, highest temperature, lowest temperature, relative humidity, atmospheric pressure, SO_2_, NO_2_, O_3_, CO, and PM_10_

§ Final model was adjusted for obesity, smoking status (current smoker compared to nonsmoker or past smoker), frequency of alcohol consumption (≥ 1 time a week compared to < 1 time a week), CCI score, relative humidity, SO_2_, NO_2_, O_3_, CO, and PM_10_ using backward selection method.

**Table S3** Crude and adjusted odd ratios (95% confidence interval) of the meteorological and pollution matter (mean of 5 days before index date) for ischemic heart disease

| Characteristics | | Odds ratio for ischemic heart disease (95% CI) | | | | | |
| --- | --- | --- | --- | --- | --- | --- | --- |
|  |  | Crude † | P-value | Adjusted †‡ | P-value | Final †§ | P-value |
| Daily mean temperature for 5 days (˚C) | | 1.00 (1.00-1.01) | 0.840 | 0.91 (0.76-1.09) | 0.310 |  |  |
| Daily highest temperature for 5 days (˚C) | | 1.00 (1.00-1.01) | 0.843 | 1.04 (0.96-1.14) | 0.348 |  |  |
| Daily lowest temperature for 5 days (˚C) | | 1.00 (1.00-1.01) | 0.800 | 1.06 (0.96-1.16) | 0.273 |  |  |
| Relative humidity for 5 days (%) | | 1.00 (0.99-1.00) | 0.287 | 1.00 (0.99-1.00) | 0.189 |  |  |
| Ambient atmospheric pressure for 5 days (hPa) | | 1.00 (0.99-1.01) | 0.632 | 1.00 (0.99-1.01) | 0.556 |  |  |
| SO_2_ for 5 days (0.01 ppm) | | 1.26 (1.01-1.57) | 0.042* | 1.59 (1.15-2.21) | 0.005* | 1.26 (1.00-1.57) | 0.047* |
| NO_2_ for 5 days (0.1 ppm) | | 0.65 (0.37-1.13) | 0.124 | 0.50 (0.21-1.18) | 0.116 |  |  |
| O_3_ for 5 days (0.01 ppm) | | 1.03 (0.97-1.08) | 0.345 | 0.97 (0.90-1.05) | 0.514 |  |  |
| CO for 5 days (ppm) | | 0.97 (0.77-1.24) | 0.820 | 0.91 (0.59-1.41) | 0.671 |  |  |
| PM_10_ for 5 days (10 μg/m^3^) | | 1.00 (0.98-1.02) | 0.920 | 1.00 (0.97-1.03) | 0.946 |  |  |

* Conditional logistic regression model, Significance at P < 0.05

† Stratified model for age, sex, income, and region of residence.

‡ Adjusted model was adjusted for obesity, smoking status (current smoker compared to nonsmoker or past smoker), frequency of alcohol consumption (≥ 1 time a week compared to < 1 time a week), CCI score, mean temperature, highest temperature, lowest temperature, relative humidity, atmospheric pressure, SO_2_, NO_2_, O_3_, CO, and PM_10_

§ Final model was adjusted for obesity, smoking status (current smoker compared to nonsmoker or past smoker), frequency of alcohol consumption (≥ 1 time a week compared to < 1 time a week), CCI score, relative humidity, SO_2_, NO_2_, O_3_, CO, and PM_10_ using backward selection method.

**Table S4** Crude and adjusted odd ratios (95% confidence interval) of the meteorological and pollution matter (mean of 10 days before index date) for ischemic heart disease

| Characteristics | | Odds ratio for ischemic heart disease (95% CI) | | | | | |
| --- | --- | --- | --- | --- | --- | --- | --- |
|  |  | Crude † | P-value | Adjusted †‡ | P-value | Final †§ | P-value |
| Mean temperature for 10 days (˚C) | | 1.00 (1.00-1.01) | 0.802 | 0.93 (0.74-1.18) | 0.561 |  |  |
| Highest temperature for 10 days (˚C) | | 1.00 (1.00-1.01) | 0.822 | 1.03 (0.92-1.15) | 0.618 |  |  |
| Lowest temperature for 10 days (˚C) | | 1.00 (1.00-1.01) | 0.775 | 1.05 (0.93-1.19) | 0.463 |  |  |
| Relative humidity for 10 days (%) | | 1.00 (0.99-1.00) | 0.231 | 0.99 (0.99-1.00) | 0.105 |  |  |
| Ambient atmospheric pressure for 10 days (hPa) | | 1.00 (0.99-1.01) | 0.652 | 1.00 (0.99-1.01) | 0.815 |  |  |
| SO_2_ for 10 days (0.01 ppm) | | 1.28 (1.01-1.61) | 0.038* | 1.63 (1.15-2.31) | 0.006* | 1.28 (1.01-1.62) | 0.040* |
| NO_2_ for 10 days (0.1 ppm) | | 0.59 (0.33-1.07) | 0.083 | 0.42 (0.17-1.04) | 0.061 |  |  |
| O_3_ for 10 days (0.01 ppm) | | 1.03 (0.98-1.09) | 0.231 | 0.97 (0.89-1.06) | 0.554 |  |  |
| CO for 10 days (ppm) | | 0.98 (0.76-1.26) | 0.873 | 0.95 (0.60-1.52) | 0.844 |  |  |
| PM_10_ for 10 days (10 μg/m^3^) | | 1.00 (0.97-1.03) | 0.966 | 1.00 (0.97-1.05) | 0.839 |  |  |

* Conditional logistic regression model, Significance at P < 0.05

† Stratified model for age, sex, income, and region of residence.

‡ Adjusted model was adjusted for obesity, smoking status (current smoker compared to nonsmoker or past smoker), frequency of alcohol consumption (≥ 1 time a week compared to < 1 time a week), CCI score, mean temperature, highest temperature, lowest temperature, relative humidity, atmospheric pressure, SO_2_, NO_2_, O_3_, CO, and PM_10_

§ Final model was adjusted for obesity, smoking status (current smoker compared to nonsmoker or past smoker), frequency of alcohol consumption (≥ 1 time a week compared to < 1 time a week), CCI score, relative humidity, SO_2_, NO_2_, O_3_, CO, and PM_10_ using backward selection method.

**Table S5** Crude and adjusted odd ratios (95% confidence interval) of the meteorological and pollution matter (mean of 15 days before index date) for ischemic heart disease

| Characteristics | | Odds ratio for ischemic heart disease (95% CI) | | | | | |
| --- | --- | --- | --- | --- | --- | --- | --- |
|  |  | Crude † | P-value | Adjusted †‡ | P-value | Final †§ | P-value |
| Mean temperature for 15 days (˚C) | | 1.00 (1.00-1.01) | 0.810 | 0.83 (0.63-1.08) | 0.156 |  |  |
| Highest temperature for 15 days (˚C) | | 1.00 (1.00-1.01) | 0.830 | 1.09 (0.96-1.24) | 0.194 |  |  |
| Lowest temperature for 15 days (˚C) | | 1.00 (1.00-1.01) | 0.761 | 1.12 (0.97-1.29) | 0.118 |  |  |
| Relative humidity for 15 days (%) | | 1.00 (0.99-1.00) | 0.278 | 0.99 (0.99-1.00) | 0.088 |  |  |
| Ambient atmospheric pressure for 15 days (hPa) | | 1.00 (0.99-1.01) | 0.660 | 1.00 (0.99-1.01) | 0.793 |  |  |
| SO_2_ for 15 days (0.01 ppm) | | 1.33 (1.05-1.69) | 0.019* | 1.71 (1.19-2.46) | 0.004* | 1.34 (1.05-1.70) | 0.018* |
| NO_2_ for 15 days (0.1 ppm) | | 0.61 (0.33-1.12) | 0.113 | 0.42 (0.17-1.08) | 0.072 |  |  |
| O_3_ for 15 days (0.01 ppm) | | 1.03 (0.98-1.09) | 0.266 | 0.98 (0.90-1.08) | 0.705 |  |  |
| CO for 15 days (ppm) | | 1.01 (0.78-1.30) | 0.970 | 0.95 (0.59-1.55) | 0.847 |  |  |
| PM_10_ for 15 days (10 μg/m^3^) | | 1.00 (0.97-1.03) | 0.885 | 1.01 (0.97-1.06) | 0.668 |  |  |

* Conditional logistic regression model, Significance at P < 0.05

† Stratified model for age, sex, income, and region of residence.

‡ Adjusted model was adjusted for obesity, smoking status (current smoker compared to nonsmoker or past smoker), frequency of alcohol consumption (≥ 1 time a week compared to < 1 time a week), CCI score, mean temperature, highest temperature, lowest temperature, relative humidity, atmospheric pressure, SO_2_, NO_2_, O_3_, CO, and PM_10_

§ Final model was adjusted for obesity, smoking status (current smoker compared to nonsmoker or past smoker), frequency of alcohol consumption (≥ 1 time a week compared to < 1 time a week), CCI score, relative humidity, SO_2_, NO_2_, O_3_, CO, and PM_10_ using backward selection method.

**Table S6** Crude and adjusted odd ratios (95% confidence interval) of the meteorological and pollution matter (mean of 60 days before index date) for ischemic heart disease

| Characteristics | | Odds ratio for ischemic heart disease (95% CI) | | | | | |
| --- | --- | --- | --- | --- | --- | --- | --- |
|  |  | Crude † | P-value | Adjusted †‡ | P-value | Final †§ | P-value |
| Mean temperature for 60 days (˚C) | | 1.00 (1.00-1.01) | 0.838 | 0.89 (0.60-1.31) | 0.544 |  |  |
| Highest temperature for 60 days (˚C) | | 1.00 (1.00-1.01) | 0.931 | 1.03 (0.85-1.24) | 0.777 |  |  |
| Lowest temperature for 60 days (˚C) | | 1.00 (1.00-1.01) | 0.756 | 1.11 (0.91-1.36) | 0.314 |  |  |
| Relative humidity for 60 days (%) | | 1.00 (0.99-1.00) | 0.288 | 0.99 (0.98-1.00) | 0.032* |  |  |
| Ambient atmospheric pressure for 60 days (hPa) | | 1.00 (0.99-1.01) | 0.714 | 1.00 (0.99-1.01) | 0.662 |  |  |
| SO_2_ for 60 days (0.01 ppm) | | 1.40 (1.08-1.82) | 0.011* | 1.76 (1.17-2.66) | 0.007* | 1.71 (1.28-2.29) | <0.001* |
| NO_2_ for 60 days (0.1 ppm) | | 0.55 (0.29-1.06) | 0.075 | 0.32 (0.11-0.91) | 0.032* | 0.32 (0.15-0.67) | 0.003* |
| O_3_ for 60 days (0.01 ppm) | | 1.05 (0.98-1.12) | 0.163 | 0.98 (0.87-1.10) | 0.675 |  |  |
| CO for 60 days (ppm) | | 0.99 (0.75-1.31) | 0.932 | 1.00 (0.57-1.76) | 0.992 |  |  |
| PM_10_ for 60 days (10 μg/m^3^) | | 1.01 (0.97-1.05) | 0.648 | 1.04 (0.98-1.11) | 0.204 |  |  |

* Conditional logistic regression model, Significance at P < 0.05

† Stratified model for age, sex, income, and region of residence.

‡ Adjusted model was adjusted for obesity, smoking status (current smoker compared to nonsmoker or past smoker), frequency of alcohol consumption (≥ 1 time a week compared to < 1 time a week), CCI score, mean temperature, highest temperature, lowest temperature, relative humidity, atmospheric pressure, SO_2_, NO_2_, O_3_, CO, and PM_10_

§ Final model was adjusted for obesity, smoking status (current smoker compared to nonsmoker or past smoker), frequency of alcohol consumption (≥ 1 time a week compared to < 1 time a week), CCI score, relative humidity, SO_2_, NO_2_, O_3_, CO, and PM_10_ using backward selection method.

**Table S7** Crude and adjusted odd ratios (95% confidence interval) of the meteorological and pollution matter (mean of 90 days before index date) for ischemic heart disease

| Characteristics | | Odds ratio for ischemic heart disease (95% CI) | | | | | |
| --- | --- | --- | --- | --- | --- | --- | --- |
|  |  | Crude † | P-value | Adjusted †‡ | P-value | Final †§ | P-value |
| Mean temperature for 90 days (˚C) | | 1.00 (1.00-1.01) | 0.822 | 0.81 (0.53-1.25) | 0.343 |  |  |
| Highest temperature for 90 days (˚C) | | 1.00 (1.00-1.01) | 0.924 | 1.06 (0.86-1.30) | 0.581 |  |  |
| Lowest temperature for 90 days (˚C) | | 1.00 (1.00-1.01) | 0.728 | 1.18 (0.94-1.47) | 0.164 |  |  |
| Relative humidity for 90 days (%) | | 1.00 (0.99-1.00) | 0.267 | 0.99 (0.98-1.00) | 0.029* |  |  |
| Ambient atmospheric pressure for 90 days (hPa) | | 1.00 (0.99-1.01) | 0.705 | 1.00 (0.99-1.01) | 0.650 |  |  |
| SO_2_ for 90 days (0.01 ppm) | | 1.39 (1.06-1.82) | 0.017* | 1.65 (1.07-2.54) | 0.022* | 1.69 (1.25-2.30) | 0.001* |
| NO_2_ for 90 days (0.1 ppm) | | 0.52 (0.27-1.02) | 0.058 | 0.30 (0.10-0.88) | 0.029* | 0.31 (0.15-0.67) | 0.003* |
| O_3_ for 90 days (0.01 ppm) | | 1.06 (0.99-1.13) | 0.104 | 0.99 (0.87-1.13) | 0.877 |  |  |
| CO for 90 days (ppm) | | 0.96 (0.72-1.28) | 0.762 | 1.04 (0.57-1.88) | 0.906 |  |  |
| PM_10_ for 90 days (10 μg/m^3^) | | 1.01 (0.97-1.05) | 0.593 | 1.06 (0.99-1.13) | 0.117 |  |  |

* Conditional logistic regression model, Significance at P < 0.05

† Stratified model for age, sex, income, and region of residence.

‡ Adjusted model was adjusted for obesity, smoking status (current smoker compared to nonsmoker or past smoker), frequency of alcohol consumption (≥ 1 time a week compared to < 1 time a week), CCI score, mean temperature, highest temperature, lowest temperature, relative humidity, atmospheric pressure, SO_2_, NO_2_, O_3_, CO, and PM_10_

§ Final model was adjusted for obesity, smoking status (current smoker compared to nonsmoker or past smoker), frequency of alcohol consumption (≥ 1 time a week compared to < 1 time a week), CCI score, relative humidity, SO_2_, NO_2_, O_3_, CO, and PM_10_ using backward selection method.

**Table S8** Crude and adjusted odd ratios (95% confidence interval) of the meteorological and pollution matter (mean of 180 days before index date) for ischemic heart disease

| Characteristics | | Odds ratio for ischemic heart disease (95% CI) | | | | | |
| --- | --- | --- | --- | --- | --- | --- | --- |
|  |  | Crude † | P-value | Adjusted †‡ | P-value | Final †§ | P-value |
| Mean temperature for 180 days (˚C) | | 1.00 (0.99-1.01) | 0.693 | 0.82 (0.48-1.41) | 0.474 |  |  |
| Highest temperature for 180 days (˚C) | | 1.00 (0.99-1.01) | 0.822 | 1.04 (0.80-1.35) | 0.765 |  |  |
| Lowest temperature for 180 days (˚C) | | 1.00 (1.00-1.01) | 0.577 | 1.19 (0.89-1.58) | 0.241 |  |  |
| Relative humidity for 180 days (%) | | 1.00 (0.99-1.00) | 0.194 | 0.98 (0.97-1.00) | 0.012* |  |  |
| Ambient atmospheric pressure for 180 days (hPa) | | 1.00 (0.99-1.01) | 0.638 | 1.00 (0.99-1.01) | 0.889 |  |  |
| SO_2_ for 180 days (0.01 ppm) | | 1.51 (1.10-2.09) | 0.012* | 1.80 (1.13-2.88) | 0.014* | 1.81 (1.28-2.57) | 0.001* |
| NO_2_ for 180 days (0.1 ppm) | | 0.41 (0.20-0.87) | 0.019* | 0.20 (0.06-0.62) | 0.005* | 0.27 (0.12-0.60) | 0.001* |
| O_3_ for 180 days (0.01 ppm) | | 1.09 (1.00-1.19) | 0.054 | 0.95 (0.81-1.10) | 0.474 |  |  |
| CO for 180 days (ppm) | | 0.86 (0.61-1.22) | 0.407 | 0.92 (0.47-1.78) | 0.797 |  |  |
| PM_10_ for 180 days (10 μg/m^3^) | | 1.01 (0.96-1.07) | 0.614 | 1.08 (1.00-1.17) | 0.051 |  |  |

* Conditional logistic regression model, Significance at P < 0.05

† Stratified model for age, sex, income, and region of residence.

‡ Adjusted model was adjusted for obesity, smoking status (current smoker compared to nonsmoker or past smoker), frequency of alcohol consumption (≥ 1 time a week compared to < 1 time a week), CCI score, mean temperature, highest temperature, lowest temperature, relative humidity, atmospheric pressure, SO_2_, NO_2_, O_3_, CO, and PM_10_

§ Final model was adjusted for obesity, smoking status (current smoker compared to nonsmoker or past smoker), frequency of alcohol consumption (≥ 1 time a week compared to < 1 time a week), CCI score, relative humidity, SO_2_, NO_2_, O_3_, CO, and PM_10_ using backward selection method.

**Table S9** Crude and adjusted odd ratios (95% confidence interval) of the meteorological and pollution matter (mean of 270 days before index date) for ischemic heart disease

| Characteristics | | Odds ratio for ischemic heart disease (95% CI) | | | | | |
| --- | --- | --- | --- | --- | --- | --- | --- |
|  |  | Crude † | P-value | Adjusted †‡ | P-value | Final †§ | P-value |
| Mean temperature for 270 days (˚C) | | 1.01 (0.99-1.02) | 0.280 | 0.89 (0.46-1.73) | 0.726 |  |  |
| Highest temperature for 270 days (˚C) | | 1.01 (0.99-1.02) | 0.430 | 1.02 (0.74-1.39) | 0.926 |  |  |
| Lowest temperature for 270 days (˚C) | | 1.01 (1.00-1.03) | 0.185 | 1.13 (0.79-1.60) | 0.504 |  |  |
| Relative humidity for 270 days (%) | | 0.99 (0.98-1.00) | 0.024* | 0.98 (0.97-0.99) | 0.003* | 0.99 (0.97-1.00) | 0.010* |
| Ambient atmospheric pressure for 270 days (hPa) | | 1.00 (0.99-1.01) | 0.500 | 1.00 (0.99-1.01) | 0.932 |  |  |
| SO_2_ for 270 days (0.01 ppm) | | 1.68 (1.15-2.47) | 0.008* | 1.82 (1.11-2.99) | 0.018* | 1.89 (1.27-2.81) | 0.002* |
| NO_2_ for 270 days (0.1 ppm) | | 0.35 (0.16-0.77) | 0.009* | 0.22 (0.07-0.73) | 0.013* | 0.23 (0.10-0.53) | 0.001* |
| O_3_ for 270 days (0.01 ppm) | | 1.20 (1.07-1.35) | 0.002* | 1.03 (0.85-1.24) | 0.771 |  |  |
| CO for 270 days (ppm) | | 0.75 (0.49-1.16) | 0.194 | 0.86 (0.43-1.74) | 0.683 |  |  |
| PM_10_ for 270 days (10 μg/m^3^) | | 1.01 (0.95-1.08) | 0.720 | 1.11 (1.01-1.21) | 0.024* |  |  |

* Conditional logistic regression model, Significance at P < 0.05

† Stratified model for age, sex, income, and region of residence.

‡ Adjusted model was adjusted for obesity, smoking status (current smoker compared to nonsmoker or past smoker), frequency of alcohol consumption (≥ 1 time a week compared to < 1 time a week), CCI score, mean temperature, highest temperature, lowest temperature, relative humidity, atmospheric pressure, SO_2_, NO_2_, O_3_, CO, and PM_10_

§ Final model was adjusted for obesity, smoking status (current smoker compared to nonsmoker or past smoker), frequency of alcohol consumption (≥ 1 time a week compared to < 1 time a week), CCI score, relative humidity, SO_2_, NO_2_, O_3_, CO, and PM_10_ using backward selection method.

**Table S10** Crude and adjusted odd ratios (95% confidence interval) of the meteorological and pollution matter (mean of 540 days before index date) for ischemic heart disease

| Characteristics | | Odds ratio for ischemic heart disease (95% CI) | | | | | |
| --- | --- | --- | --- | --- | --- | --- | --- |
|  |  | Crude † | P-value | Adjusted †‡ | P-value | Final †§ | P-value |
| Mean temperature for 540 days (˚C) | | 1.01 (0.99-1.04) | 0.176 | 0.79 (0.38-1.65) | 0.529 |  |  |
| Highest temperature for 540 days (˚C) | | 1.01 (0.99-1.03) | 0.307 | 1.09 (0.77-1.54) | 0.632 |  |  |
| Lowest temperature for 540 days (˚C) | | 1.02 (1.00-1.04) | 0.103 | 1.18 (0.80-1.74) | 0.408 |  |  |
| Relative humidity for 540 days (%) | | 0.98 (0.97-1.00) | 0.021* | 0.98 (0.97-0.99) | 0.006* | 0.98 (0.97-1.00) | 0.020* |
| Ambient atmospheric pressure for 540 days (hPa) | | 1.00 (0.99-1.01) | 0.455 | 1.00 (0.99-1.01) | 0.874 |  |  |
| SO_2_ for 540 days (0.01 ppm) | | 1.78 (1.19-2.67) | 0.005* | 1.79 (1.05-3.03) | 0.031* | 1.78 (1.18-2.67) | 0.006* |
| NO_2_ for 540 days (0.1 ppm) | | 0.39 (0.17-0.85) | 0.019* | 0.31 (0.09-1.12) | 0.073 |  |  |
| O_3_ for 540 days (0.01 ppm) | | 1.26 (1.11-1.43) | <0.001* | 1.14 (0.90-1.44) | 0.285 | 1.25 (1.10-1.43) | 0.001* |
| CO for 540 days (ppm) | | 0.73 (0.47-1.14) | 0.170 | 0.85 (0.41-1.76) | 0.653 |  |  |
| PM_10_ for 540 days (10 μg/m^3^) | | 1.01 (0.94-1.08) | 0.797 | 1.13 (1.02-1.25) | 0.015* |  |  |

* Conditional logistic regression model, Significance at P < 0.05

† Stratified model for age, sex, income, and region of residence.

‡ Adjusted model was adjusted for obesity, smoking status (current smoker compared to nonsmoker or past smoker), frequency of alcohol consumption (≥ 1 time a week compared to < 1 time a week), CCI score, mean temperature, highest temperature, lowest temperature, relative humidity, atmospheric pressure, SO_2_, NO_2_, O_3_, CO, and PM_10_

§ Final model was adjusted for obesity, smoking status (current smoker compared to nonsmoker or past smoker), frequency of alcohol consumption (≥ 1 time a week compared to < 1 time a week), CCI score, relative humidity, SO_2_, NO_2_, O_3_, CO, and PM_10_ using backward selection method.

**Table S11** Crude and adjusted odd ratios (95% confidence interval) of the meteorological and pollution matter (mean of 730 days before index date) for ischemic heart disease

| Characteristics | | Odds ratio for ischemic heart disease (95% CI) | | | | | |
| --- | --- | --- | --- | --- | --- | --- | --- |
|  |  | Crude † | P-value | Adjusted †‡ | P-value | Final †§ | P-value |
| Mean temperature for 730 days (˚C) | | 1.08 (1.03-1.12) | 0.001* | 0.74 (0.31-1.75) | 0.492 |  |  |
| Highest temperature for 730 days (˚C) | | 1.07 (1.02-1.12) | 0.006* | 1.12 (0.77-1.64) | 0.557 |  |  |
| Lowest temperature for 730 days (˚C) | | 1.06 (1.02-1.10) | 0.001* | 1.18 (0.75-1.86) | 0.483 |  |  |
| Relative humidity for 730 days (%) | | 0.98 (0.96-0.99) | 0.004* | 0.97 (0.95-0.99) | 0.007* | 0.98 (0.96-1.00) | 0.008* |
| Ambient atmospheric pressure for 730 days (hPa) | | 1.00 (0.99-1.01) | 0.399 | 1.00 (0.99-1.02) | 0.793 |  |  |
| SO_2_ for 730 days (0.01 ppm) | | 1.72 (1.13-2.62) | 0.012* | 1.56 (0.90-2.69) | 0.110 |  |  |
| NO_2_ for 730 days (0.1 ppm) | | 0.38 (0.17-0.85) | 0.019* | 0.29 (0.05-1.77) | 0.180 |  |  |
| O_3_ for 730 days (0.01 ppm) | | 1.33 (1.16-1.53) | <0.001* | 1.42 (1.05-1.92) | 0.023* | 1.70 (1.40-2.07) | <0.001* |
| CO for 730 days (ppm) | | 0.69 (0.44-1.10) | 0.117 | 0.83 (0.39-1.77) | 0.626 |  |  |
| PM_10_ for 730 days (10 μg/m^3^) | | 1.01 (0.94-1.08) | 0.897 | 1.21 (1.08-1.36) | 0.001* | 1.21 (1.10-1.34) | <0.001* |

* Conditional logistic regression model, Significance at P < 0.05

† Stratified model for age, sex, income, and region of residence.

‡ Adjusted model was adjusted for obesity, smoking status (current smoker compared to nonsmoker or past smoker), frequency of alcohol consumption (≥ 1 time a week compared to < 1 time a week), CCI score, mean temperature, highest temperature, lowest temperature, relative humidity, atmospheric pressure, SO_2_, NO_2_, O_3_, CO, and PM_10_

§ Final model was adjusted for obesity, smoking status (current smoker compared to nonsmoker or past smoker), frequency of alcohol consumption (≥ 1 time a week compared to < 1 time a week), CCI score, relative humidity, SO_2_, NO_2_, O_3_, CO, and PM_10_ using backward selection method.

**Table S12** Akaike information criterion and Baysian information criterion of the pollution matters in the final model logistic regression for ischemic heart disease

| Meteorological and pollution matters | Final *† | |
| --- | --- | --- |
|  | AIC | BIC |
| Meteorological and pollution matters for 730 days | 9,935.64 | 10,008.49 |
| Meteorological and pollution matters for 540 days | 9,948.16 | 10,021.01 |
| Meteorological and pollution matters for 365 days | 9,934.52 | 10,014.66 |
| Meteorological and pollution matters for 270 days | 9,949.08 | 10,021.93 |
| Meteorological and pollution matters for 180 days | 9,953.53 | 10,019.10 |
| Meteorological and pollution matters for 90 days | 9,955.14 | 10,020.71 |
| Meteorological and pollution matters for 60 days | 9,954.18 | 10,019.75 |
| Meteorological and pollution matters for 30 days | 9,961.43 | 10,019.71 |
| Meteorological and pollution matters for 15 days | 9,961.74 | 10,020.02 |
| Meteorological and pollution matters for 10 days | 9,963.06 | 10,021.34 |
| Meteorological and pollution matters for 5 days | 9,963.35 | 10,021.63 |
| Meteorological and pollution matters for 3 days | 9,962.29 | 10,020.57 |

Abbreviations: AIC, Akaike information criterion; BIC, Baysian information criterion

* Stratified model for age, sex, income, and region of residence.

† Final model was adjusted for obesity, smoking status (current smoker compared to nonsmoker or past smoker), frequency of alcohol consumption (≥ 1 time a week compared to < 1 time a week), CCI score, relative humidity, SO_2_, NO_2_, O_3_, CO, and PM_10_ using backward selection method.

**Table S13** Meteorological and air pollution data for 3 days, 5 days, 10 days, 15 days, 2 months (60 days), 3 months (90 days), 6 months (180 days), 9 months (270 days), 18 months (540 days), and 2 years (730 days)

| Characteristics | | | Total participants | | |
| --- | --- | --- | --- | --- | --- |
|  | |  | IHD | Control | P-value |
| Meteorological and air pollution data (mean, SD) | | | |  |  |
|  | Mean temperature for 3 days (˚C) | | 12.0 (9.8) | 11.9 (9.8) | 0.853 |
|  | Highest temperature for 3 days (˚C) | | 17.2 (9.7) | 17.2 (9.8) | 0.840 |
|  | Lowest temperature for 3 days (˚C) | | 7.5 (10.2) | 7.4 (10.2) | 0.855 |
|  | Relative humidity for 3 days (%) | | 65.0 (13.2) | 65.3 (13.0) | 0.237 |
|  | Ambient atmospheric pressure for 3 days (hPa) | | 1006.3 (8.4) | 1006.4 (8.2) | 0.633 |
|  | SO_2_ for 3 days (ppb) | | 5.7 (2.2) | 5.6 (2.3) | 0.026* |
|  | NO_2_ for 3 days (ppb) | | 23.4 (9.7) | 23.7 (9.9) | 0.173 |
|  | O_3_ for 3 days (ppb) | | 23.4 (9.7) | 23.2 (9.9) | 0.357 |
|  | CO for 3 days (ppb) | | 564.5 (210.7) | 566.7 (213.2) | 0.670 |
|  | PM_10_ for 3 days (μg/m^3^) | | 51.4 (22.7) | 51.6 (23.7) | 0.786 |
|  | Mean temperature for 5 days (˚C) | | 12.0 (9.7) | 12.0 (9.7) | 0.845 |
|  | Highest temperature for 5 days (˚C) | | 17.3 (9.6) | 17.2 (9.6) | 0.848 |
|  | Lowest temperature for 5 days (˚C) | | 7.5 (10.1) | 7.4 (10.1) | 0.807 |
|  | Relative humidity for 5 days (%) | | 65.1 (12.2) | 65.4 (12.0) | 0.318 |
|  | Ambient atmospheric pressure for 5 days (hPa) | | 1006.3 (8.1) | 1006.4 (7.9) | 0.644 |
|  | SO_2_ for 5 days (ppb) | | 5.7 (2.1) | 5.6 (2.2) | 0.047* |
|  | NO_2_ for 5 days (ppb) | | 23.5 (9.0) | 23.8 (9.2) | 0.153 |
|  | O_3_ for 5 days (ppb) | | 23.4 (9.2) | 23.2 (9.4) | 0.364 |
|  | CO for 5 days (ppb) | | 566.8 (201.6) | 567.9 (201.6) | 0.825 |
|  | PM_10_ for 5 days (μg/m^3^) | | 51.4 (19.7) | 51.5 (20.3) | 0.922 |
|  | Mean temperature for 10 days (˚C) | | 12.0 (9.6) | 12.0 (9.6) | 0.808 |
|  | Highest temperature for 10 days (˚C) | | 17.3 (9.4) | 17.3 (9.4) | 0.827 |
|  | Lowest temperature for 10 days (˚C) | | 7.5 (10.0) | 7.5 (10.0) | 0.783 |
|  | Relative humidity for 10 days (%) | | 65.0 (11.2) | 65.3 (11.0) | 0.264 |
|  | Ambient atmospheric pressure for 10 days (hPa) | | 1006.3 (7.8) | 1006.4 (7.7) | 0.663 |
|  | SO_2_ for 10 days (ppb) | | 5.7 (2.0) | 5.6 (2.1) | 0.042* |
|  | NO_2_ for 10 days (ppb) | | 23.5 (8.5) | 23.8 (8.7) | 0.109 |
|  | O_3_ for 10 days (ppb) | | 23.4 (8.8) | 23.2 (8.9) | 0.247 |
|  | CO for 10 days (ppb) | | 571.0 (196.6) | 571.8 (193.2) | 0.876 |
|  | PM_10_ for 10 days (μg/m^3^) | | 51.9 (17.5) | 51.9 (17.8) | 0.967 |
|  | Mean temperature for 15 days (˚C) | | 17.4 (9.4) | 17.3 (9.4) | 0.816 |
|  | Highest temperature for 15 days (˚C) | | 7.6 (10.0) | 7.5 (10.0) | 0.835 |
|  | Lowest temperature for 15 days (˚C) | | 65.1 (10.6) | 65.3 (10.6) | 0.769 |
|  | Relative humidity for 15 days (%) | | 1006.3 (7.7) | 1006.4 (7.6) | 0.313 |
|  | Ambient atmospheric pressure for 15 days (hPa) | | 5.7 (2.0) | 5.6 (2.0) | 0.671 |
|  | SO_2_ for 15 days (ppb) | | 23.5 (8.4) | 23.8 (8.4) | 0.021* |
|  | NO_2_ for 15 days (ppb) | | 23.4 (8.5) | 23.1 (8.6) | 0.143 |
|  | O_3_ for 15 days (ppb) | | 571.0 (190.1) | 570.9 (186.8) | 0.284 |
|  | CO for 15 days (ppb) | | 52.0 (16.2) | 51.9 (16.5) | 0.970 |
|  | PM_10_ for 15 days (μg/m^3^) | | 17.4 (9.4) | 17.3 (9.4) | 0.888 |
|  | Mean temperature for 60 days (˚C) | | 12.3 (9.2) | 12.3 (9.2) | 0.843 |
|  | Highest temperature for 60 days (˚C) | | 17.6 (9.0) | 17.6 (8.9) | 0.933 |
|  | Lowest temperature for 60 days (˚C) | | 7.9 (9.7) | 7.8 (9.6) | 0.764 |
|  | Relative humidity for 60 days (%) | | 65.5 (9.5) | 65.7 (9.5) | 0.329 |
|  | Ambient atmospheric pressure for 60 days (hPa) | | 1006.2 (7.3) | 1006.3 (7.3) | 0.723 |
|  | SO_2_ for 60 days (ppb) | | 5.7 (1.8) | 5.6 (1.8) | 0.012* |
|  | NO_2_ for 60 days (ppb) | | 23.4 (7.9) | 23.7 (8.0) | 0.102 |
|  | O_3_ for 60 days (ppb) | | 23.2 (7.7) | 23.0 (7.7) | 0.179 |
|  | CO for 60 days (ppb) | | 570.8 (177.0) | 571.2 (172.6) | 0.933 |
|  | PM_10_ for 60 days (μg/m^3^) | | 51.8 (13.0) | 51.6 (13.2) | 0.656 |
|  | Mean temperature for 90 days (˚C) | | 12.5 (8.8) | 12.5 (8.7) | 0.827 |
|  | Highest temperature for 90 days (˚C) | | 17.8 (8.5) | 17.8 (8.5) | 0.926 |
|  | Lowest temperature for 90 days (˚C) | | 8.1 (9.2) | 8.0 (9.2) | 0.737 |
|  | Relative humidity for 90 days (%) | | 65.7 (9.0) | 65.9 (9.0) | 0.313 |
|  | Ambient atmospheric pressure for 90 days (hPa) | | 1006.1 (7.1) | 1006.2 (7.1) | 0.715 |
|  | SO_2_ for 90 days (ppb) | | 5.7 (1.8) | 5.6 (1.8) | 0.020* |
|  | NO_2_ for 90 days (ppb) | | 23.3 (7.8) | 23.7 (7.8) | 0.083 |
|  | O_3_ for 90 days (ppb) | | 23.2 (7.1) | 22.9 (7.1) | 0.118 |
|  | CO for 90 days (ppb) | | 569.3 (170.7) | 570.5 (166.6) | 0.768 |
|  | PM_10_ for 90 days (μg/m^3^) | | 51.5 (12.1) | 51.4 (12.2) | 0.602 |
|  | Mean temperature for 180 days (˚C) | | 13.1 (6.3) | 13.0 (6.4) | 0.702 |
|  | Highest temperature for 180 days (˚C) | | 18.3 (6.1) | 18.3 (6.2) | 0.827 |
|  | Lowest temperature for 180 days (˚C) | | 8.6 (6.7) | 8.6 (6.7) | 0.592 |
|  | Relative humidity for 180 days (%) | | 66.0 (7.0) | 66.2 (7.0) | 0.256 |
|  | Ambient atmospheric pressure for 180 days (hPa) | | 1005.7 (6.0) | 1005.8 (6.0) | 0.650 |
|  | SO_2_ for 180 days (ppb) | | 5.6 (1.5) | 5.5 (1.5) | 0.013* |
|  | NO_2_ for 180 days (ppb) | | 23.1 (7.1) | 23.5 (7.2) | 0.035* |
|  | O_3_ for 180 days (ppb) | | 23.5 (5.8) | 23.3 (5.7) | 0.066 |
|  | CO for 180 days (ppb) | | 562.7 (141.8) | 565.4 (138.9) | 0.419 |
|  | PM_10_ for 180 days (μg/m^3^) | | 51.5 (9.5) | 51.4 (9.6) | 0.626 |
|  | Mean temperature for 270 days (˚C) | | 13.1 (3.2) | 13.0 (3.2) | 0.301 |
|  | Highest temperature for 270 days (˚C) | | 18.4 (3.1) | 18.3 (3.1) | 0.442 |
|  | Lowest temperature for 270 days (˚C) | | 8.7 (3.5) | 8.6 (3.5) | 0.217 |
|  | Relative humidity for 270 days (%) | | 65.9 (5.2) | 66.1 (5.2) | 0.073 |
|  | Ambient atmospheric pressure for 270 days (hPa) | | 1005.6 (4.9) | 1005.7 (4.9) | 0.518 |
|  | SO_2_ for 270 days (ppb) | | 5.6 (1.2) | 5.5 (1.2) | 0.009* |
|  | NO_2_ for 270 days (ppb) | | 23.1 (6.6) | 23.5 (6.8) | 0.018* |
|  | O_3_ for 270 days (ppb) | | 23.8 (4.4) | 23.4 (4.4) | 0.003* |
|  | CO for 270 days (ppb) | | 561.8 (116.3) | 565.2 (113.6) | 0.207 |
|  | PM_10_ for 270 days (μg/m^3^) | | 51.8 (7.7) | 51.7 (7.8) | 0.730 |
|  | Mean temperature for 540 days (˚C) | | 12.9 (2.4) | 12.8 (2.4) | 0.198 |
|  | Highest temperature for 540 days (˚C) | | 18.1 (2.3) | 18.1 (2.2) | 0.318 |
|  | Lowest temperature for 540 days (˚C) | | 8.5 (2.8) | 8.4 (2.7) | 0.140 |
|  | Relative humidity for 540 days (%) | | 65.8 (4.7) | 66.0 (4.7) | 0.081 |
|  | Ambient atmospheric pressure for 540 days (hPa) | | 1005.8 (4.8) | 1005.8 (4.7) | 0.474 |
|  | SO_2_ for 540 days (ppb) | | 5.6 (1.2) | 5.5 (1.2) | 0.006* |
|  | NO_2_ for 540 days (ppb) | | 23.3 (6.6) | 23.7 (6.8) | 0.037* |
|  | O_3_ for 540 days (ppb) | | 23.4 (3.9) | 23.1 (3.9) | 0.001* |
|  | CO for 540 days (ppb) | | 570.2 (114.8) | 573.8 (110.1) | 0.195 |
|  | PM_10_ for 540 days (μg/m^3^) | | 52.3 (7.4) | 52.3 (7.4) | 0.805 |
|  | Mean temperature for 730 days (˚C) | | 12.8 (1.2) | 12.8 (1.2) | 0.004* |
|  | Highest temperature for 730 days (˚C) | | 18.1 (1.0) | 18.0 (1.0) | 0.006* |
|  | Lowest temperature for 730 days (˚C) | | 8.4 (1.7) | 8.3 (1.7) | 0.010* |
|  | Relative humidity for 730 days (%) | | 65.8 (4.3) | 66.0 (4.3) | 0.039* |
|  | Ambient atmospheric pressure for 730 days (hPa) | | 1005.8 (4.6) | 1005.9 (4.5) | 0.420 |
|  | SO_2_ for 730 days (ppb) | | 5.6 (1.1) | 5.6 (1.1) | 0.012* |
|  | NO_2_ for 730 days (ppb) | | 23.4 (6.5) | 23.8 (6.8) | 0.033* |
|  | O_3_ for 730 days (ppb) | | 23.4 (3.6) | 23.1 (3.7) | <0.001* |
|  | CO for 730 days (ppb) | | 574.9 (110.6) | 578.8 (105.6) | 0.142 |
|  | PM_10_ for 730 days (μg/m^3^) | | 52.9 (7.0) | 52.9 (7.1) | 0.901 |

Abbreviations: IHD, ischemic heart disease; BMI, body mass index (kg/m^2^); ppb, Parts per billion; ppm, Part per million (= 1,000 ppb); SD, standard deviation

* Independent T-test. Significance at P < 0.05

**Table S14.** Pearson’s correlation coefficients (r) between each of meteorological and air pollution variables for 30 days

|  | Mean temperature | Highest temperature | Lowest temperature | Relative humidity | pressure | SO_2_ | NO_2_ | O_3_ | CO | PM_10_ |
| --- | --- | --- | --- | --- | --- | --- | --- | --- | --- | --- |
| Mean temperature | 1.000 | 0.993* | 0.995* | 0.619* | -0.670* | -0.618* | -0.489* | 0.430* | -0.698* | -0.479* |
| Highest temperature | 0.993* | 1.000 | 0.977* | 0.605* | -0.673* | -0.620* | -0.488* | 0.457* | -0.681* | -0.441* |
| Lowest temperature | 0.995* | 0.977* | 1.000 | 0.633* | -0.655* | -0.609* | -0.482* | 0.387* | -0.702* | -0.512* |
| Relative humidity | 0.619* | 0.605* | 0.633* | 1.000* | -0.450* | -0.404* | -0.444* | 0.013 | -0.334* | -0.475* |
| Pressure | -0.670* | -0.673* | -0.655* | -0.450* | 1.000 | 0.42*0 | 0.467* | -0.455* | 0.382* | 0.196* |
| SO_2_ | -0.618* | -0.620* | -0.609* | -0.404* | 0.420* | 1.000 | 0.466* | -0.259* | 0.686* | 0.503* |
| NO_2_ | -0.489* | -0.488* | -0.482* | -0.444* | 0.467* | 0.466* | 1.000 | -0.482* | 0.553* | 0.528* |
| O_3_ | 0.430* | 0.457* | 0.387* | 0.013 | -0.455* | -0.25*9 | -0.482* | 1.000 | -0.505* | 0.006 |
| CO | -0.698* | -0.681* | -0.702* | -0.334* | 0.382* | 0.686* | 0.553* | -0.505* | 1.000 | 0.530* |
| PM_10_ | -0.479* | -0.441* | -0.512* | -0.475* | 0.196* | 0.503* | 0.528* | 0.006 | 0.530* | 1.000 |

* Pearson’s correlation coefficient analysis, Significance at P < 0.05

Pressure: Ambient atmospheric pressure

**Table S15.** Pearson’s correlation coefficients (r) between each of meteorological and air pollution variables for 365 days

|  | Mean temperature | Highest temperature | Lowest temperature | Relative humidity | Pressure | SO_2_ | NO_2_ | O_3_ | CO | PM_10_ |
| --- | --- | --- | --- | --- | --- | --- | --- | --- | --- | --- |
| Mean temperature | 1.000 | 0.806* | 0.948* | -0.384* | 0.368* | -0.001 | -0.268* | 0.354* | -0.455* | -0.357* |
| Highest temperature | 0.806* | 1.000 | 0.581* | -0.169* | 0.323* | -0.050* | -0.435* | 0.368* | -0.264* | -0.244* |
| Lowest temperature | 0.948* | 0.581* | 1.000 | -0.433* | 0.344* | 0.046* | -0.123* | 0.279* | -0.489* | -0.345* |
| Relative humidity | -0.384* | -0.169* | -0.433* | 1.000 | -0.063* | 0.030* | -0.385* | 0.225* | 0.069* | -0.013 |
| Pressure | 0.368* | 0.323* | 0.344* | -0.063* | 1.000 | 0.039* | 0.224* | -0.067* | -0.275* | -0.085* |
| SO_2_ | -0.001 | -0.050* | 0.046* | 0.030* | 0.039* | 1.000 | 0.228* | -0.043* | 0.343* | 0.275* |
| NO_2_ | -0.268* | -0.435* | -0.123* | -0.385* | 0.224* | 0.228* | 1.000 | -0.731* | 0.341* | 0.545* |
| O_3_ | 0.354* | 0.368* | 0.279* | 0.225* | -0.067* | -0.043* | -0.731* | 1.000 | -0.481* | -0.622* |
| CO | -0.455* | -0.264* | -0.489* | 0.069* | -0.275* | 0.343* | 0.341* | -0.481* | 1.000 | 0.574* |
| PM_10_ | -0.357* | -0.244* | -0.345* | -0.013 | -0.085* | 0.275* | 0.545* | -0.622* | 0.574* | 1.000 |

* Pearson’s correlation coefficient analysis, Significance at P < 0.05

Pressure: Ambient atmospheric pressure
